# Dexmedetomidine post‐treatment attenuates cardiac ischaemia/reperfusion injury by inhibiting apoptosis through HIF‐1α signalling

**DOI:** 10.1111/jcmm.14795

**Published:** 2019-11-03

**Authors:** Ke Peng, Wei‐rong Chen, Fan Xia, Hong Liu, Xiao‐wen Meng, Juan Zhang, Hua‐yue Liu, Zheng‐yuan Xia, Fu‐hai Ji

**Affiliations:** ^1^ Department of Anesthesiology First Affiliated Hospital of Soochow University Suzhou China; ^2^ Department of Anesthesiology Soochow University Affiliated Children's Hospital Suzhou China; ^3^ Department of Anesthesiology and Pain Medicine University of California Davis Health Sacramento CA USA

**Keywords:** cardiac apoptosis, cardioprotection, dexmedetomidine, HIF‐1α, hypoxia/reoxygenation, ischaemia/reperfusion

## Abstract

Hypoxia‐inducible factor 1α (HIF‐1α) plays a critical role in the apoptotic process during cardiac ischaemia/reperfusion (I/R) injury. This study aimed to investigate whether post‐treatment with dexmedetomidine (DEX) could protect against I/R‐induced cardiac apoptosis in vivo and in vitro via regulating HIF‐1α signalling pathway. Rat myocardial I/R was induced by occluding the left anterior descending artery for 30 minutes followed by 6‐hours reperfusion, and cardiomyocyte hypoxia/reoxygenation (H/R) was induced by oxygen‐glucose deprivation for 6 hours followed by 3‐hours reoxygenation. Dexmedetomidine administration at the beginning of reperfusion or reoxygenation attenuated I/R‐induced myocardial injury or H/R‐induced cell death, alleviated mitochondrial dysfunction, reduced the number of apoptotic cardiomyocytes, inhibited the activation of HIF‐1α and modulated the expressions of apoptosis‐related proteins including BCL‐2, BAX, BNIP3, cleaved caspase‐3 and cleaved PARP. Conversely, the HIF‐1α prolyl hydroxylase‐2 inhibitor IOX2 partly blocked DEX‐mediated cardioprotection both in vivo and in vitro. Mechanistically, DEX down‐regulated HIF‐1α expression at the post‐transcriptional level and inhibited the transcriptional activation of the target gene *BNIP3*. Post‐treatment with DEX protects against cardiac I/R injury in vivo and H/R injury in vitro. These effects are, at least in part, mediated via the inhibition of cell apoptosis by targeting HIF‐1α signalling.

## 1 INTRODUCTION

Although myocardial reperfusion is fundamental to the salvage of viable myocardium, the restoring coronary blood flow paradoxically leads to the death of cardiomyocytes—a phenomenon termed ischaemia/reperfusion (I/R) injury.^1^, ^2^ During procedures such as cardiopulmonary bypass and percutaneous coronary intervention, I/R injury is inevitable.^3^ In recent years, several ischaemic conditioning and pharmacological strategies to prevent I/R injury have been examined; however, the translation from experimental findings to clinical application is extremely difficult.^4^, ^5^

Apoptosis has been found to underlie myocardial I/R injury and infarction.^6^ Apoptotic process is initiated shortly after myocardial ischaemia and is greatly enhanced during reperfusion.^7^ Hypoxia‐inducible factor 1α (HIF‐1α) is a critical transcription factor and a key regulator in the process of hypoxia‐induced apoptosis.^8^ Depending on the severity of hypoxia, HIF‐1α either promotes cell survival by facilitating adaptation to low‐oxygen environments, or it induces apoptosis by activating pro‐apoptotic signalling.^8^

The α2‐adrenergic agonist dexmedetomidine (DEX) produces various beneficial effects in surgical patients, including sympatholytic, sedative, analgesic and opioid‐sparing effects.^9^ Our previous study suggested that DEX use during cardiac surgery was correlated with a lower post‐operative mortality rate and fewer complications.^10^ Animal studies showed that DEX pre‐treatment attenuated myocardial I/R injury, in part, by inhibiting inflammatory responses.^11^, ^12^

Compared with pre‐treatment, DEX administration at the onset of reperfusion (post‐treatment) is more applicable in clinical practice. However, the underlying mechanism of DEX post‐treatment during cardiac I/R injury is unclear. Given the essential role of HIF‐1α in the I/R process, we hypothesized that post‐treatment with DEX could inhibit cardiac apoptosis after I/R in rats and after hypoxia/reoxygenation (H/R) in cardiomyocytes by regulating the expression of HIF‐1α.

## 2 MATERIALS AND METHODS

### 2.1 Animals and cells

Animal experimental protocol was approved by the Ethics Committee for Animal Experimentation of Soochow University (#2016‐064). The experimental procedures complied with the Guide for the Care and Use of Laboratory Animals (US National Institutes of Health). Adult male Sprague‐Dawley rats (8‐10 weeks old; 300‐350 g) and neonatal rats (1‐2 days old) were obtained from the Experimental Animal Centre of Soochow University, Suzhou, China. The rats received standard rodent food and water in a controlled environment (12‐hours light/dark cycle; room temperature, 22°C; 4‐5 rats per cage).

Neonatal rat cardiomyocyte cultures were prepared according to the method previously described.^13^, ^14^ In brief, rat hearts were harvested and kept in a sterile phosphate‐buffered saline solution on ice. The ventricles were minced and digested five times with 0.1% type II collagenase for 5 minutes each time at 37°C. The supernatants were collected in Dulbecco modified Eagle medium‐F12 (DMEM‐F12) supplemented with 15% foetal bovine serum (FBS). Fibroblasts were separated by incubation for 2 hours and extraction of non‐adherent cells. Cells were seeded onto plates and cultured in media with 1% 5‐bromodeoxyuridine in a humidified incubator containing 5% CO_2_ at 37°C. The culture media were replaced the next day, and the cells were used for experiments 3‐4 days later.

### 2.2 Drugs and reagents

Dexmedetomidine (Jiangsu Nhwa Pharmaceutical Co. Ltd.) in phosphate‑buffered saline (PBS); IOX2 (ApexBio) in 5% dimethyl sulfoxide (DMSO); type II collagenase, 5‐bromodeoxyuridine, sodium pentobarbital, 2,3,5‐triphenyltetrazoliumchloride (Sigma); DMEM‐F12, non‐serum DMEM, FBS (Gibco); protease inhibitor cocktail (Roche); anti‐HIF‐1α, anti‐BCL‐2, anti‐BNIP3 (abcam); anti‐caspase‐3, anti‐β‐tubulin (Cell Signalling Technology); anti‐BAX (Cell Signalling Technology and Ybscience); anti‐PARP‐1 (Santa Cruz Biotechnology); RT‐PCR primers (Sanggon); Trizol (Invitrogen); 5X All‐in‐One RT MasterMix, DNA fectin Plus, all vectors used in luciferase reporter gene assay (abm).

### 2.3 I/R and H/R models

The myocardial I/R model was established as previously described.^12^, ^15^ First, the rats were intraperitoneally anaesthetized with 40 mg/kg sodium pentobarbital. The rats then underwent intubation, ventilation (tidal volume, 5 mL/kg; 80 times/min), left thoracotomy and left anterior descending (LAD) artery ligation ~2 mm below the left auricle. The LAD occlusion was maintained for 30 minutes of ischaemia, and then, the ligature was released for reperfusion of 2, 6 and 24 hours. Model establishment was confirmed by S‐T segment elevation and myocardial blanching. A 24G intravenous catheter was inserted into the right jugular vein for DEX infusion. Throughout the procedure, the body temperature of the rats was maintained at 37°C by using a heating pad and a rectal thermometer. After the experiment, the animals were sacrificed after intraperitoneal pentobarbital 100 mg/kg.

To establish the in vitro H/R model, the neonatal rat cardiomyocytes were incubated in an anaerobic Plexiglas chamber (Billups‐Rothenberg) containing 95% N_2_ and 5% CO_2_ at 37°C. Prior to hypoxia, the culture media were replaced with non‐glucose and non‐serum DMEM. For reoxygenation, the cells were transferred to FBS‐containing DMEM under normal conditions.

### 2.4 Experimental protocols

**Figure 1:**
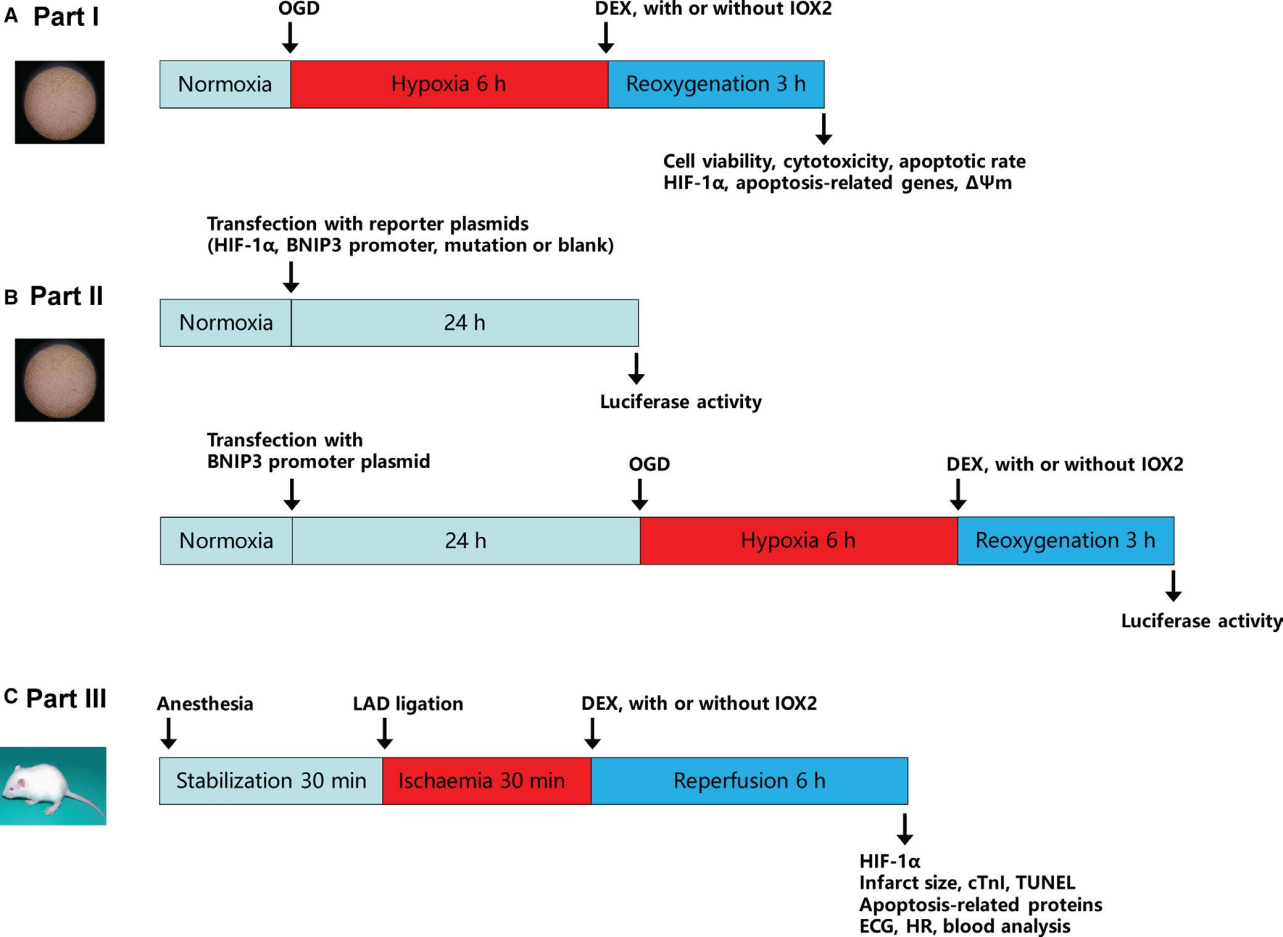
Experimental protocols. A, Part I: neonatal rat cardiomyocytes were subjected to hypoxia/reoxygenation. B, Part II: cells were transfected with reporter plasmids and luciferase activity was assessed. C, Part III: rats underwent myocardial ischaemia/reperfusion. OGD, oxygen‐glucose deprivation; DEX, dexmedetomidine; LAD, left anterior descending; ∆Ψm, mitochondrial membrane potential; ECG, electrocardiography; cTnI, serum cardiac troponin I; and HR, heart rate

An investigator who was not involved in the subsequent study performed randomization using a computer‐generated table and prepared the study solutions. The investigators who carried out the following experiments and data analyses were blinded to the group allocation. The experimental protocols are presented in Figure 1.

1. To evaluate the possible cytotoxicity of DEX, cell viability was measured after treatments with 0.1, 1, 10 and 50 μM DEX for 24 hours in normal conditions.
2. To observe the time course of cell survival during H/R, cell viability was assessed after exposure to hypoxia for 3, 6 and 12 hours followed by reoxygenation for 3, 6, 12 and 24 hours.
3. Cell viability and cytotoxicity were measured after 0.1, 1, and 10 μM DEX treatments in cells subjected to H/R.
4. DEX 1 μM was added to the culture media at the beginning of reoxygenation. The apoptosis rate and the expression of HIF‐1α and BAX were determined.
5. To explore the role of HIF‐1α in the cardioprotective effects of DEX, we tested the effects of 50 μM IOX2, a selective and potent inhibitor of HIF‐1α prolyl hydroxylase‐2 (PHD2),^16^, ^17^, ^18^ on cell viability, the mitochondrial apoptotic pathway, and the expression of HIF‐1α and the apoptosis‐related proteins including B‐cell lymphoma‐2 (BCL‐2), Bcl‐2 associated X protein (BAX), Bcl‐2/adenovirus E1B 19kD‐interacting protein 3 (BNIP3), cleaved caspase‐3 and poly ADP‐ribose polymerase 1 (PARP1).
6. To further investigate the underlying mechanisms, we evaluated the HIF‐1α and BNIP3 mRNA levels and the transcriptional activation of the *BNIP3* promoter by HIF‐1α in cardiomyocytes.
7. In rats, HIF‐1α protein levels were measured at 2, 6 and 24 hours of reperfusion to analyse the time course of HIF‐1α expression during I/R.
8. To examine the effects of DEX on myocardial injury, HIF‐1α and apoptosis, DEX (6 μg/kg/h × 10 minutes + 0.7 μg/kg/h × 15 minutes) was administered intravenously at the beginning of reperfusion. Serum cardiac troponin I (cTnI), myocardial apoptosis index, infract size, and the expression of HIF‐1α and apoptosis‐related proteins were analysed. IOX2 25 mg/kg was injected intraperitoneally prior to DEX administration.^16^ The sham rats underwent chest open without LAD ligation and received normal saline infusion. The doses of DEX 12, 19 and IOX2^16^, ^17^ use in this study were based on our preliminary experiments and previous studies.

### 2.5 Electrocardiography and blood analysis

Throughout the animal experiment, electrocardiographic (ECG) changes were monitored using a biological signal‐processing system (MedLab). Heart rate was recorded at the baseline, at 15 and 30 minutes of ischaemia and at 15, 30 and 60 minutes of reperfusion. At 6 hours of reperfusion, blood samples were taken from the abdominal aorta and the pH, partial pressures of oxygen (PaO_2_) and carbon dioxide (PaCO_2_), arterial oxygen saturation (SaO_2_), haemoglobin (Hb), haematocrit (Hct), Na^+^, K^+^, Ca^2+^, Cl^−^, HCO_3_^−^ and base excess (BE) were measured using a blood‐gas analyzer (Radiometer).

### 2.6 Enzyme‐linked immunosorbent assay

Serum cTnI levels were quantified using a commercial kit (Life Diagnostics) according to the manufacturer's instructions. We measured the absorbance values at 450 nm by using the SpectraMax190 plate reader (MD) and determined the sample concentrations by using a standard curve.

### 2.7 TUNEL assay

Myocardial apoptosis was detected by TUNEL assays (Roche) according to the manufacturer's instructions. Myocardial tissue slices were counterstained with 4′,6‐diamidino‐2‐phenylindole (DAPI). The total myocardial cell nuclei and TUNEL‐positive nuclei were counted in four random and non‐overlapping fields per slice. The apoptosis index was defined as the ratio of TUNEL‐positive cells to the total number of cells. The cells were imaged using the DM2500 fluorescence microscope (Leica), and the images were analysed with ImageJ (NIH).

### 2.8 Infarct size

Following I/R, we re‐occluded the LAD and injected 2% Evans blue dye into the aorta. The heart was excised, frozen and transversely sectioned into five 2‐mm‐thick slices. Next, all slices were stained using 1% 2,3,5‐triphenyltetrazoliumchloride at 37°C for 30 minutes and digitally photographed. The images were analysed with ImageJ. The infarct area (IA) was expressed as a percentage of the total area at risk (AAR): IA/AAR × 100%.

### 2.9 Cell viability and lactate dehydrogenase activity

Cell viability was evaluated by using the Cell Counting Kit‐8 (CCK‐8) assay (Beyotime), and cytotoxicity was quantified using the lactate dehydrogenase (LDH) activity assay (Beyotime) according to the manufacturer's instructions. We measured the absorbance values at 490 nm by using the SpectraMax190 plate reader. Three technical replicates were tested, and the average value was calculated for each sample.

### 2.10 Flow cytometry

The cell apoptosis rate was measured by using an annexin V‐fluorescein isothiocyanate/propidium iodide apoptosis kit (BD Biosciences) according to the manufacturer's instructions. We analysed the cellular fluorescence with the FACSCalibur™ flow cytometer (BD Biosciences). Three technical replicates were applied for each sample.

### 2.11 Mitochondrial membrane potential

Mitochondrial membrane potential (∆Ψm) changes were detected by JC‐1 staining (Beyotime) according to the manufacturer's instructions. In healthy cells with polarized inner membranes, JC‐1 accumulates as aggregates, showing red fluorescence. In apoptotic cells with ∆Ψm dissipation, cytosolic JC‐1 monomers show green fluorescence. The ratio of JC‐1 monomers to aggregates was calculated in four random and non‐overlapping fields per slice using the DM2500 fluorescence microscope and analysed with ImageJ.

### 2.12 Immunoblotting

Cardiac tissues and cardiomyocytes were homogenized in lysis buffer supplemented with the cOmplete™ Protease Inhibitor Cocktail. Next, we separated the proteins thus obtained using sodium dodecyl sulphate‐polyacrylamide gel electrophoresis on 10%‐12% gels. We transferred the separated proteins to nitrocellulose membranes, which were then blocked and incubated overnight at 4°C with the following primary antibodies: anti‐HIF‐1α, 1:500 (abcam); anti‐BAX, 1:1000 (Cell Signalling Technology); anti‐BCL‐2, 1:500 (abcam); anti‐BNIP3, 1:1000 (abcam); anti‐caspase‐3, 1:1000 (Cell Signalling Technology); anti‐PARP1, 1:500 (Santa Cruz Technology); and anti‐β‐tubulin, 1:1000 (Cell Signalling Technology). After this, the membranes were incubated for 1 hour at room temperature with rat anti‐mouse or goat anti‐rabbit secondary antibodies. Three replicates were tested for each sample. Finally, we visualized the blots by using chemiluminescence and a luminescent imaging workstation (Tanon 5200).

### 2.13 Immunofluorescence staining

Cells grown on coverslips were fixed with 4% paraformaldehyde, permeabilized with 0.3% Triton X‐100, blocked and incubated overnight at 4°C with the following primary antibodies: anti‐HIF‐1α, 1:200 (abcam); anti‐BAX, 1:200 (Ybscience); and anti‐α‐SMA, 1:200 (abcam). This was followed by incubation with the appropriate secondary antibodies for 1 hour at room temperature. We counterstained the cell nuclei with DAPI and observed and analysed the stained cells using the DM2500 fluorescence microscope and ImageJ.

### 2.14 Quantitative reverse transcription polymerase chain reaction assay

We extracted total RNA with Trizol, and reverse transcribed 1 µg of the extracted total RNA using the 5X All‐in‐One RT MasterMix. Real‐time quantitative reverse transcription polymerase chain reaction (RT‐PCR) assays were performed with the CFX96™ Touch system (Bio‐Rad) and the following specific primers: HIF‐1α, 5′‐TGAGGACACGAGCTGCCTCT‐3′ (forward) and 5′‐GTGTCATCGCTGCCGAAGT‐3′ (reverse); BNIP3, 5′‐GCTACCTCTCAGTGGTCACTTCC‐3′ (forward) and 5′‐TGCTGAAGTGCAGTTCTACCCA‐3′ (reverse); and β‐tubulin, 5′‐TGTCACCAACTGGGACGATA‐3′ (forward) and 5′‐GGGGTGTTGAAGGTCTCAAA‐3′ (reverse). Three technical replicates were applied for each sample. We assessed gene abundance using the 2^−ΔΔ^Ct method.

### 2.15 Transient transfections and dual luciferase reporter gene assay

First, neonatal rat cardiomyocytes were cotransfected with a reporter plasmid (pLenti‐miniCMV‐RenLuc‐BNIP3 promoter‐Luc‐SV40‐GFP‐2A‐Puro vector) containing the *BNIP3* promoter and the *Renilla* luciferase or a BN*IP3* promoter mutation vector, and a HIF‐1α lentiviral vector (pLenti‐GIII‐CMV‐GFP‐2A‐Puro) for overexpression or a blank vector (2 µg for each vector) using DNA fectin Plus for 24 hours. The cells were then harvested for the luciferase activity analyses. Next, neonatal rat cardiomyocytes were transfected with 2 µg reporter plasmid for 24 hours, followed by incubation under either normal or H/R conditions with or without DEX.

At the end of the transfection, we observed the cells using the DM2500 fluorescence microscope. To measure the luciferase activity, we used the Firefly & Renilla Luciferase Single Tube Assay Kit (Biotium) and the GloMax^®^ 96 Microplate Luminometer (Promega). We normalized the firefly luciferase luminescence activity to the *Renilla* luciferase activity.

### 2.16 Statistical analysis

Data are expressed as mean ± standard error of the mean (SEM). Within‐group differences were evaluated using one‐way or two‐way analysis of variance (ANOVA) followed by Dunnett test. GraphPad Prism (version 7.0) was used for all statistical analyses. *P* < .05 indicated statistically significant differences.

## 3 RESULTS

### 3.1 DEX alleviated H/R injury, inhibited apoptosis and reduced HIF‐1α and BAX expression in neonatal rat cardiomyocytes

**Figure 2:**
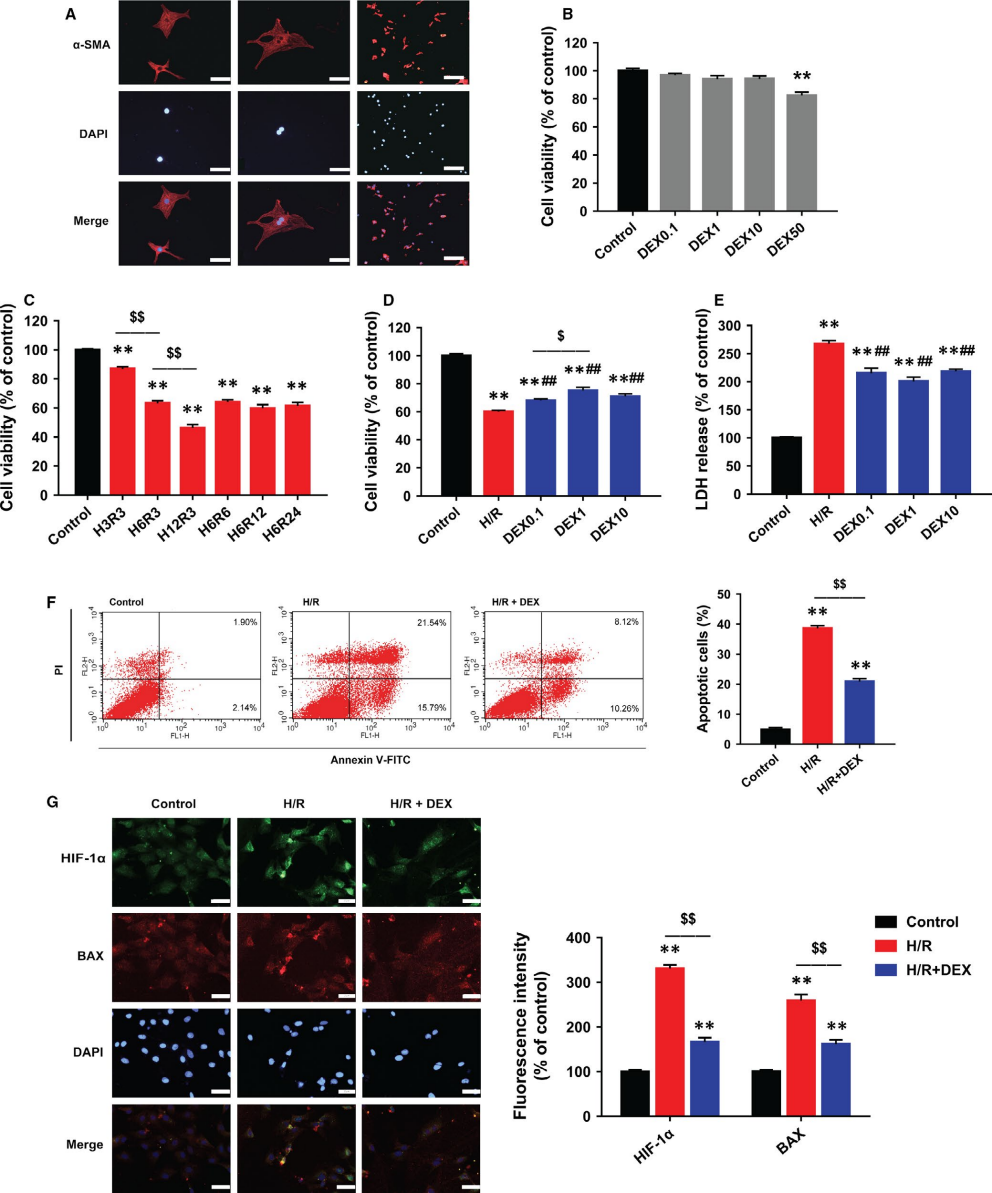
DEX post‐treatment alleviated H/R‐induced injury, inhibited apoptosis and reduced HIF‐1α and BAX expression in neonatal rat cardiomyocytes. A, Immunostaining of α‐SMA showing purified cardiomyocytes. Scale bars: 50 (left and middle rows) and 200 µm (right row). B, CCK‐8 assay showing that DEX 0.1‐10 µM had no cytotoxicity. C, CCK‐8 assay showing significant decreases in cell viability after different durations of H/R (eg H3R3 indicates 3 hours of hypoxia followed by 3‐hours of reoxygenation). D, CCK‐8 assay showing the effects of DEX post‐treatment on cell viability. E, LDH assay showing the effects of DEX on cytotoxicity. F, Flow cytometric assessment of annexin V‐FITC/PI staining showing that DEX reduced apoptotic cells. G, Immunofluorescence showing that DEX inhibited the expression of HIF‐1α (green) and BAX (red). Scale bar: 30 µm. n = 6. **P < .01 vs control; ##*P* < .01 vs H/R; $*P* < .05, $$*P* < .01 for the comparisons shown

The purified neonatal rat cardiomyocytes are shown in Figure 2A. Incubation with DEX 0.1‐10 µM did not significantly change cell viability, but DEX 50 µM resulted in decreased cell viability (Figure 2B). The maximum tolerated concentration of DEX in clinical use is 15 ng/mL (equivalent to 75 nM).^14^, ^20^ Thus, DEX is not cytotoxic to cultured neonatal rat cardiomyocytes at the clinically relevant concentration.

Hypoxia/reoxygenation exposure significantly decreased cell viability (Figure 2C). After analysing the time course of cell survival, we selected 6‐hours hypoxia followed by 3‐hours reoxygenation, which led to a 40% decrease in cell viability, for the subsequent experiments. Next, the effects of 0.1, 1 and 10 µM of DEX were evaluated. We found that 1 µM DEX led to the highest cell viability (Figure 2D) and lowest LDH release (Figure 2E).

Hypoxia/reoxygenation markedly increased the apoptosis rate, whereas post‐treatment with 1 µM DEX reduced the apoptosis rate (Figure 2F). H/R exposure also resulted in the subcellular co‐localization and high expression of HIF‐1α and BAX, which was effectively attenuated by DEX (Figure 2G).

### 3.2 DEX protected cardiomyocytes against H/R‐induced apoptosis by targeting HIF‐1α

**Figure 3:**
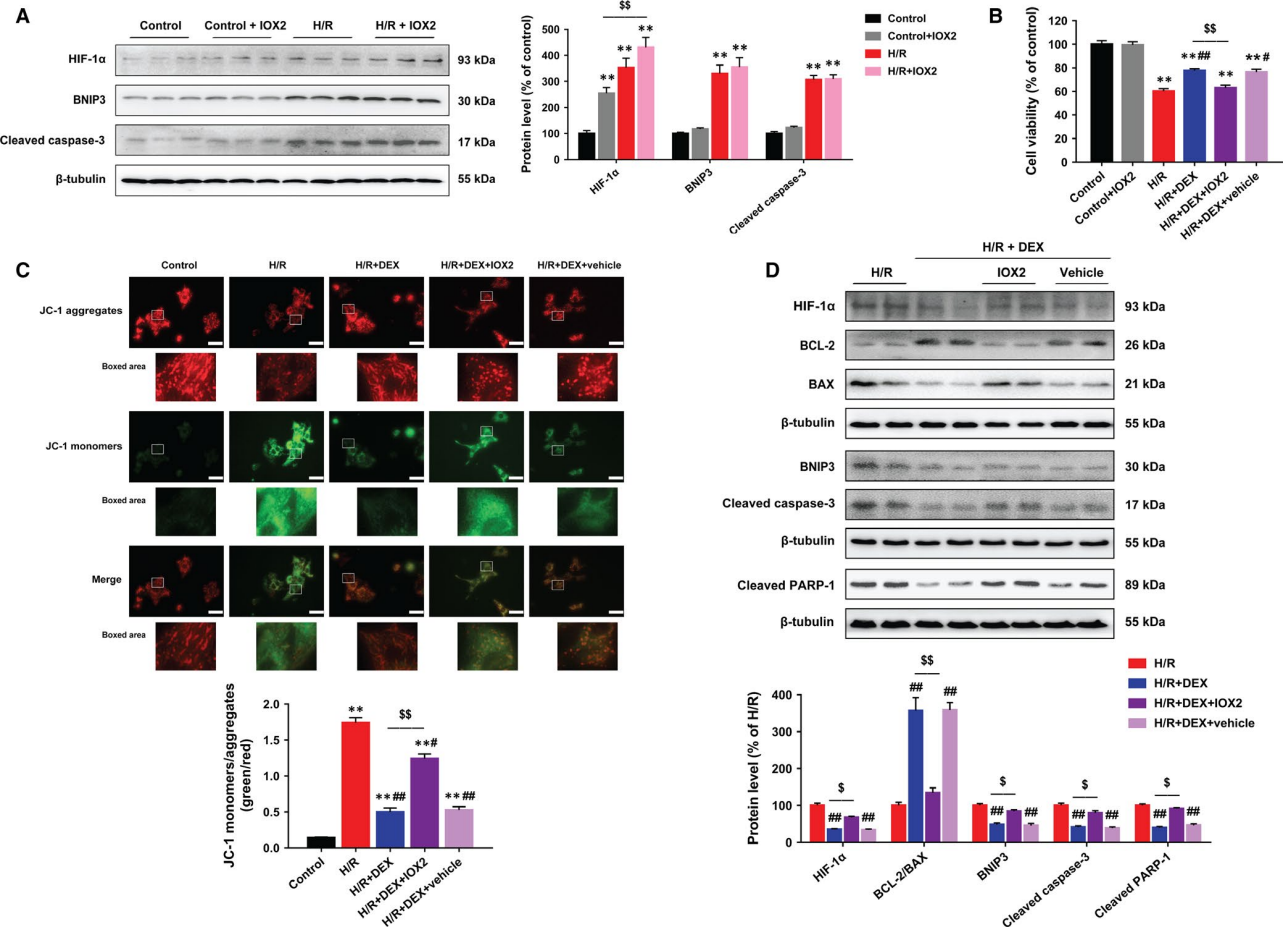
IOX2 abolished the effects of DEX post‐treatment on HIF‐1α, apoptosis‐related proteins and mitochondrial membrane potential in neonatal rat cardiomyocytes. A, Immunoblotting showing that IOX2 stabilized HIF‐1α protein expression without affecting BNIP3 and cleaved caspase‐3 protein levels. B, CCK‐8 assay showing no significant cytotoxicity associated with IOX2 and that the effects of DEX on cell viability were reversed by IOX2. C, Immunofluorescent staining of JC‐1 showing that DEX restored the ratio of JC‐1 aggregates in normal mitochondria (red) to JC‐1 monomers in dysfunctional mitochondria (green), which was partly blocked by IOX2. Scale bar: 30 µm. D, Immunoblotting showing that IOX2 partly reversed the effects of DEX treatment on HIF‐1α, BCL‐2, BAX, BNIP3, cleaved caspase‐3 and cleaved PARP‐1 protein levels. n = 6. **P < .01 vs control; #*P* < .05, ##*P* < .01 vs H/R; $*P* < .05, $$*P* < .01 for the comparisons shown

IOX2, a potent and selective PHD2 inhibitor, stabilized HIF‐1α protein expression without significant adverse effects on cell viability or apoptosis‐related proteins, including BNIP3 and cleaved caspase‐3 (Figure 3A,B). However, IOX2 treatment significantly abolished the improvement of cell viability by DEX (Figure 3B).

To investigate the effects of DEX on mitochondrial function during H/R, we detected changes in ∆Ψm by using JC‐1 staining. H/R exposure resulted in remarkable △Ψm dissipation in cells, as indicated by the increased ratio of green/red immunofluorescence intensity. Dexmedetomidine treatment attenuated H/R‐induced △Ψm dissipation, and this effect was partly blocked by IOX2 (Figure 3C). Furthermore, DEX treatment reduced HIF‐1α, BAX, BNIP3, cleaved caspase‐3 and cleaved PARP‐1 protein levels and restored BCL‐2 protein expression and the ratio of BCL‐2 to BAX in cells subjected to H/R, which was partially reversed by IOX2 (Figure 3D).

### 3.3 DEX blocked HIF‐1α‐induced transcriptional activation of BNIP3 expression in neonatal rat cardiomyocytes

**Figure 4:**
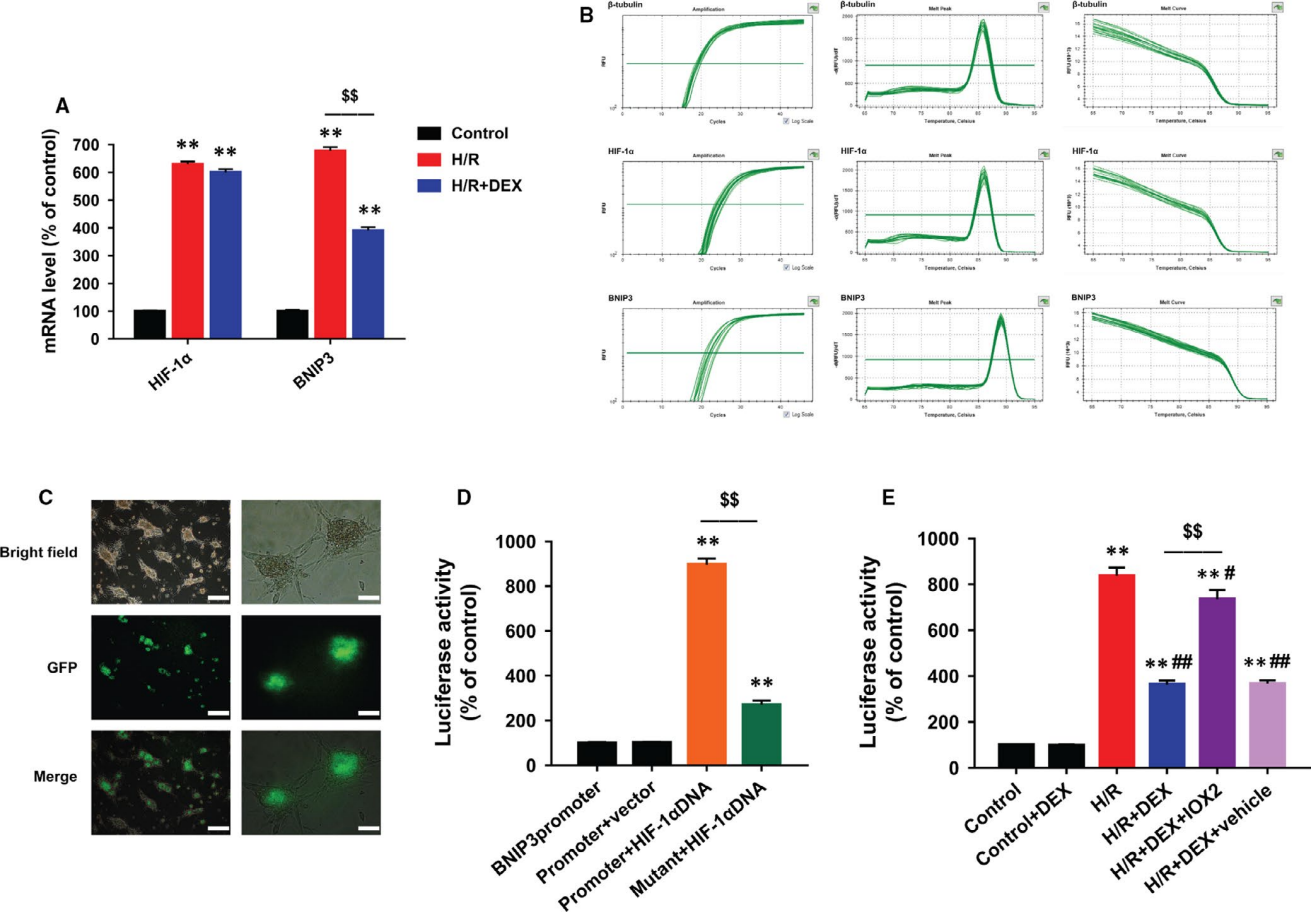
DEX inhibited HIF‐1α‐induced transcriptional activation of BNIP3 expression. A, Quantitative RT‐PCR showing that DEX reduced BNIP3 mRNA, but not HIF‐1α mRNA. B, Amplification, melt peak and melt curve of three genes in RT‐PCR. C, High green fluorescence showing that the cells were effectively transfected. Scale bars: 100 (left row) and 50 µm (right row). D, Dual luciferase reporter gene assay showing that HIF‐1α DNA led to significantly high luciferase activity, which was reduced after transfection with the *BNIP3* promoter mutation vector. E, Dual luciferase reporter gene assay showing that DEX blocked the activity of the BNIP3 promoter in the presence of hypoxia‐response elements, which was reversed by IOX2. n = 6. **P < .01 vs control (or BNIP3promoter); #*P* < .05, ##*P* < .01 vs H/R; $$*P* < .01 for the comparisons shown

Exposure of neonatal rat cardiomyocytes to H/R markedly elevated the mRNA expression of both HIF‐1α and its target gene *BNIP3*. However, DEX treatment only reduced the levels of BNIP3 mRNA, but not HIF‐1α mRNA (Figure 4A). The amplification, melt peak and melt curve of products of RT‐PCR reactions confirmed the reliability of the results (Figure 4B).

At the end of the transfection, high green fluorescence indicated that the cells were effectively transfected (Figure 4C). The cells with HIF‐1α overexpression showed significantly higher luciferase activity than the control or blank vector groups, while the luciferase activity was reduced in the *BNIP3* promoter mutant group (Figure 4D). Moreover, the luciferase reporter gene assay showed that DEX treatment reduced the luciferase‐elicited fluorescence in cells subjected to H/R, which was partly reversed by IOX2 (Figure 4E). These results confirmed that DEX blocked the HIF‐1α‐induced transcriptional activation of BNIP3 expression.

### 3.4 DEX alleviated myocardial apoptosis and I/R injury in rats

**Figure 5:**
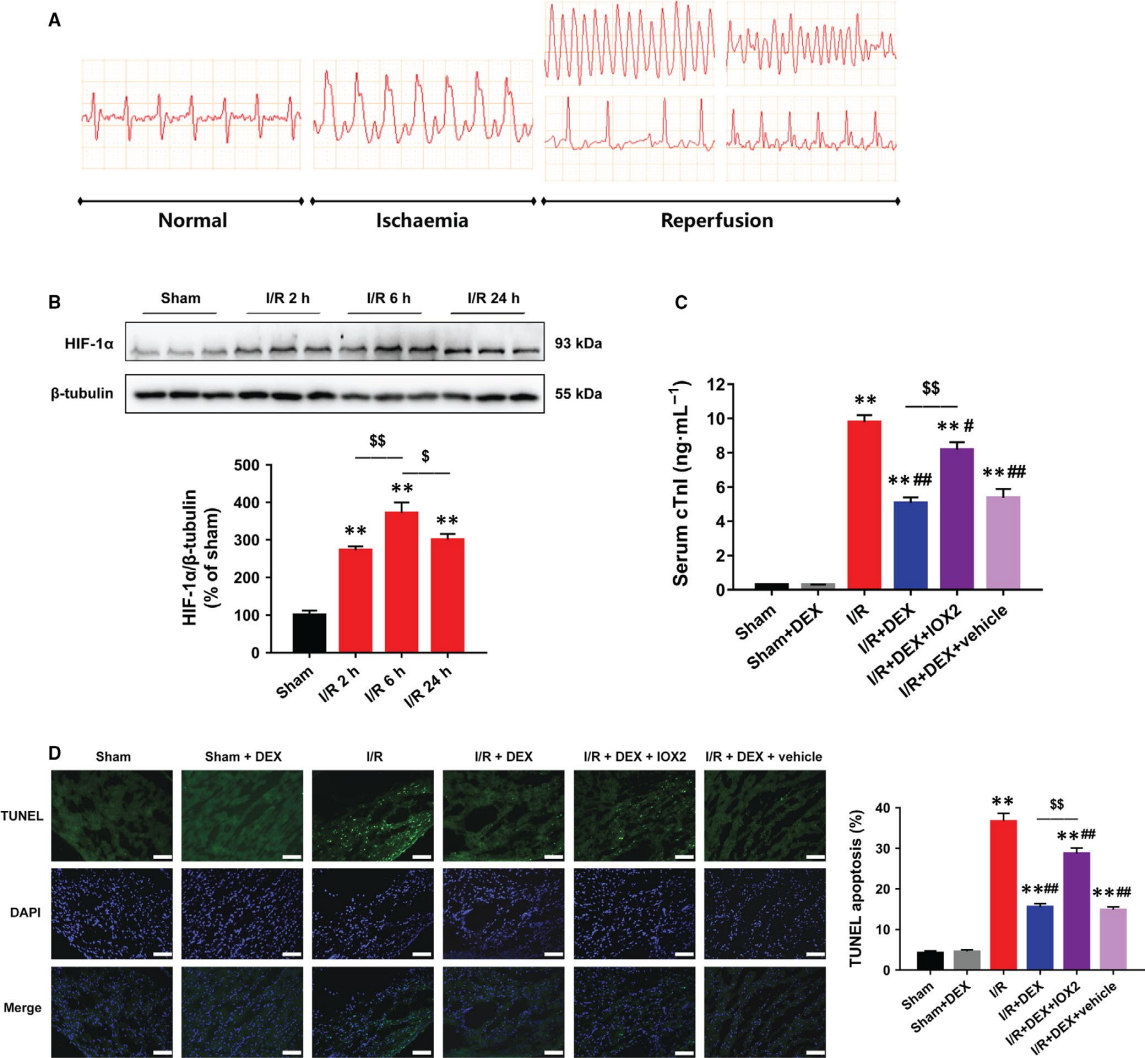
DEX alleviated myocardial I/R injury and apoptosis in rats. A, ECG patterns in normal conditions, ischaemia and reperfusion phase. B, Immunoblotting showing the time course of HIF‐1α protein expression. C, ELISA showing that DEX post‐treatment reduced the I/R‐induced increase in serum cTnI, which was reversed by IOX2. D, TUNEL assay showing that DEX reduced myocardial apoptosis after I/R, which was diminished by IOX2. Scale bar: 200 µm. n = 6. **P < .01 vs sham; #*P* < .05, ##*P* < .01 vs I/R; $*P* < .05, $$*P* < .01 for the comparisons shown

The I/R procedures resulted in remarkable S‐T segment elevation during ischaemia and arrhythmias during reperfusion (Figure 5A). Dexmedetomidine did not significantly change S‐T segment elevation or the subsequent arrhythmias. Nine animals died prior to the experimental end‐point due to blood loss, myocardial infarction or severe arrhythmias (two in I/R group, two in I/R + IOX2 group, two in IR + DEX + IOX2 group, one in I/R + DEX group, one in IR + DEX + vehicle group and one in Sham group).

During I/R, the HIF‐1α protein level peaked at 6 hours after reperfusion (Figure 5B). We selected 30 minutes ischaemia followed by 6‐hours reperfusion for the subsequent experiments. The I/R rats showed significant myocardial damage, as evidenced by increased serum cTnI levels, which was effectively reduced by DEX (Figure 5C). TUNEL assays were performed to assess DNA fragmentation in apoptotic cells. Significantly, fewer TUNEL‐positive cells were observed after DEX treatment, indicating that DEX ameliorated I/R‐induced DNA fragmentation (Figure 5D). However, these protective effects of DEX were abolished by IOX2.

During I/R, the heart rate was recorded at the baseline, 15 and 30 minutes after ischaemia, and 15, 30 and 60 minutes after reperfusion. The heart rate decreased slightly but not significantly after DEX administration (Table S1).

### 3.5 DEX protected against I/R‐induced myocardial infarction and apoptosis by targeting HIF‐1α

**Figure 6:**
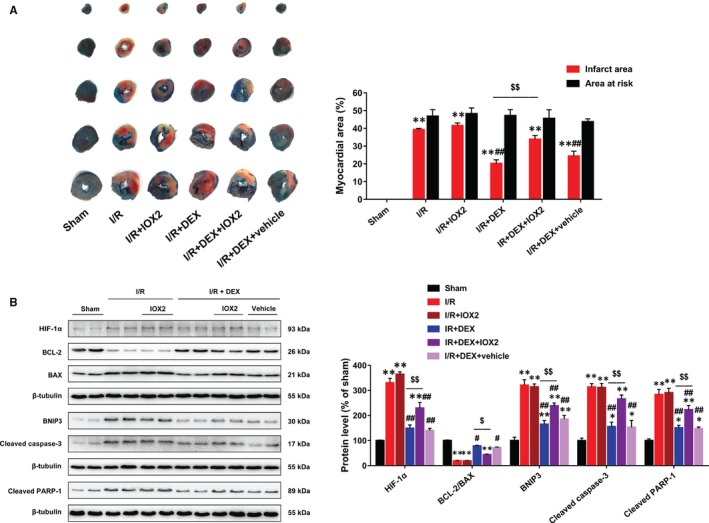
IOX2 diminished the effects of DEX post‐treatment on infarct size, HIF‐1α and apoptosis‐related proteins in rats. A, Evans blue/TTC staining showing that DEX reduced myocardial infarct size after I/R, which was abolished by IOX2. B, Immunoblotting showing that IOX2 blocked the effects of DEX on HIF‐1α, BAX, BCL‐2, BNIP3, cleaved caspase‐3 and cleaved PARP‐1 proteins. n = 6. *P < .05, **P < .01 vs sham; #*P* < .05, ##*P* < .01 vs I/R; $*P* < .05, $$*P* < .01 for the comparisons shown

Myocardial infarct size was significantly larger in the I/R group rats than in the sham group rats, and IOX2 treatment did not further increase the infarct size (Figure 6A). Dexmedetomidine treatment notably reduced infarct size, and this effect was partly diminished by IOX2. Dexmedetomidine also reduced the levels of the HIF‐1α, BAX, BNIP3, cleaved caspase‐3 and cleaved PARP‐1 proteins and increased the BCL‐2 protein level and the ratio of BCL‐2 to BAX, while IOX2 treatment partly abolished these effects (Figure 6B).

Blood analyses showed that all parameters including pH, PaO_2_, PaCO_2_, SaO_2_, Hb, Hct, Na^+^, K^+^, Ca^2+^, Cl^−^, HCO_3_^−^ and BE were within the normal range at 6 hours of reperfusion. Compared with the sham group, the I/R group rats showed decreases in PaCO_2_, HCO_3_^−^ and BE levels, but the differences among the groups were not significant (Table S2).

## 4 DISCUSSION

This study demonstrates that DEX post‐treatment conferred cardioprotection on rat hearts and cardiomyocytes, as evidenced by improved serum cTnI levels (or cell viability and LDH release), alleviated mitochondrial dysfunction, reduced DNA fragmentation (or apoptosis rate), decreased myocardial infarct size and down‐regulated protein expressions of apoptosis‐related genes. These protective effects of DEX were partly abolished by inhibiting HIF‐1α degradation with IOX2. Furthermore, this is the first study to demonstrate that DEX inhibits the HIF‐1α‐induced activation of the target gene *BNIP3* at the post‐transcriptional level.

HIF‐1α, a master regulator of the hypoxia response, is induced by hypoxia and regulates the expression of a large range of target genes. Under normal conditions, HIF‐1α protein is hydroxylated by PHDs, ubiquitinated and degraded by 26S proteasomes.^21^ During hypoxia, the hydroxylation process is blocked, and HIF‐1α accumulates to exert its transcriptional activities.^22^ The severity of hypoxia determines whether cells adapt to hypoxic stress or become apoptotic.^8^ Some studies have suggested that HIF‐1α is a fundamental element of the intrinsic survival signalling to protect against I/R injury.^23^, ^24^, ^25^ Specifically, they showed that I/R‐induced myocardial injury is alleviated by interventions that up‐regulate HIF‐1α expression. However, high HIF‐1α expression can also initiate a sequence of events that lead to apoptotic cell death.^8^ Under this condition, treatments that inhibited the HIF‐1α activity reduced I/R and H/R injuries in rat hearts and cardiomyocytes, respectively,^26^, ^27^, ^28^ which is in line with our results. A recent study also revealed that miR‐210 reduces the renal tubular cell apoptosis that occurs in response to hypoxia by suppressing HIF‐1α pathway activation.^29^ The inconsistency between the pro‐survival and the pro‐apoptotic effects of HIF‐1α may be attributable to differences in cell environments and the severity of hypoxia.

The protective effects of DEX against myocardial I/R injury have been observed in various animal models. In isolated rat hearts, DEX administration before ischaemia reduced coronary blood flow but improved infarct size, and this effect was reversed by yohimbine (an α‐2 adrenergic antagonist).^30^ In another study, DEX pre‐treatment activated Erk 1/2, Akt and eNOS, improved myocardial function and decreased infarct size after myocardial I/R both in vivo and ex vivo.^19^ In grave scalding rats, DEX protected against myocardial apoptosis.^31^ In pigs, intracoronary DEX infusion reduced reperfusion‐induced ventricular arrhythmias and suppressed plasma norepinephrine concentrations, indicating that DEX provided cardioprotection via a direct action on the myocardium.^32^ Of note, DEX pre‐treatment was found to protect isolated rat hearts and adult rat cardiomyocytes via the activation of eNOS/NO signalling, but this effect required the interaction of DEX‐pre‐treated endothelial cells.^33^ However, our findings on cardiomyocytes suggest that DEX may directly act on the cells. Recently, our studies showed that DEX pre‐treatment attenuated I/R‐induced cardiac injury by inhibiting inflammation.^11^, ^12^, ^13^ In addition to its cardioprotective benefits, DEX has been found to attenuate I/R injury in other vital organs, including the brain, liver, kidney, lungs and spinal cord.^34^, ^35^, ^36^, ^37^, ^38^

Few studies have investigated the effects of DEX post‐treatment; however, administration at the onset of reperfusion is more applicable in clinical practice. A previous study failed to show the protective effect of DEX post‐treatment against myocardial injury.^39^ However, isolated rat hearts were used in that study, and only myocardial infarct size was measured and the authors were not able to show any possible mechanism. Another study reported that the post‐ischaemic use of DEX protected the heart against I/R through PI3K/Akt‑dependent signalling, but this study did not investigate the mechanism at the cellular level.^40^ In the mouse brain, DEX post‐treatment attenuated I/R injury by inhibiting neuronal autophagy.^41^ Based on our current findings, DEX may be used for cardioprotection at the initiation of reperfusion at the clinical settings.

This study has several limitations. First, we did not conduct cardiac‐function tests to investigate the beneficial role of DEX in rats. Nevertheless, our recent study has shown that DEX improved cardiac function in isolated hearts during I/R injury.^11^ Next, no survival benefit of DEX was shown in our animals. A much larger sample size may be required to detect a difference in survival outcomes. Last, it may still be difficult to extrapolate these animal data to humans due to the species differences. Therefore, more clinical studies are needed to confirm DEX's cardioprotection.

**Figure 7:**
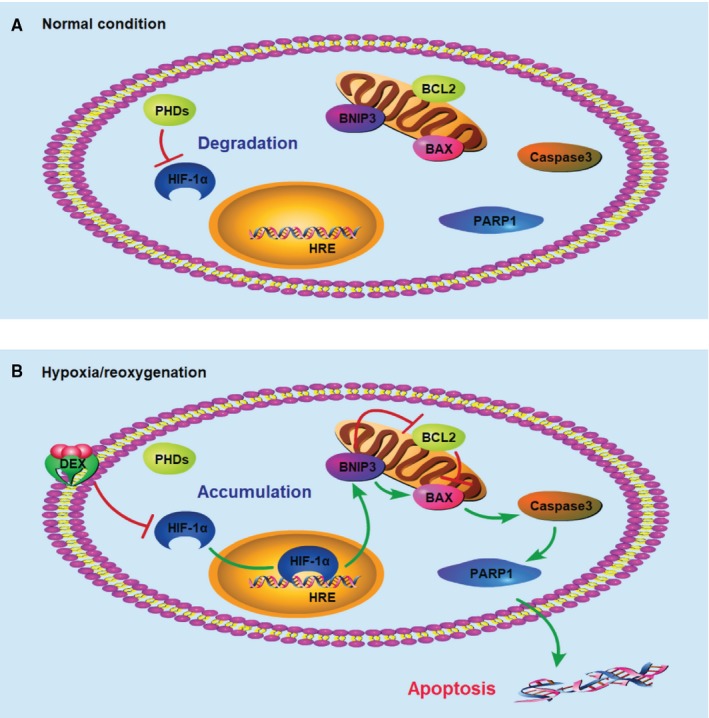
Schematic mechanism of the cardioprotective effects of DEX through targeting HIF‐1α. A, Under normal conditions, HIF‐1α protein is degraded by PHDs. B, Under H/R conditions, HIF‐1α protein accumulates and exerts transcriptional activities. DEX down‐regulates the expression of HIF‐1α at the post‐transcriptional level and inhibits the transcriptional activation of *BNIP3*. DEX, dexmedetomidine; PHD, prolyl hydroxylase; and HRE, hypoxia reaction element

In conclusion, the protective effects of DEX against rat myocardial I/R and cardiomyocyte H/R are mediated, at least in part, by the inhibition of apoptosis via the regulation of HIF‐1α signalling (Figure 7). These findings reveal a potential cardioprotective mechanism of DEX, which provides a basis for therapeutic strategies to improve outcomes in patients at risk for myocardial I/R injury.

## CONFLICT OF INTEREST

The authors have no conflicts of interest.

## AUTHOR CONTRIBUTIONS

KP and FJ designed the study. KP, WC and FX conducted the experiments and analysed the data. HL and KP drafted the manuscript. ZX and FJ helped revise the manuscript. XM helped conduct the study and analyse the data. JZ and HL helped conduct the study and wrote the manuscript. All authors contributed to revising the manuscript and approved the final version.

## Data Availability

The data that support the findings of this study are available from the corresponding author upon reasonable request.
