# Supplementary material for: Dexmedetomidine post‐treatment attenuates cardiac ischaemia/reperfusion injury by inhibiting apoptosis through HIF‐1α signalling
Source: J Cell Mol Med. 2019 Nov 3;24(1):850–61. doi: 10.1111/jcmm.14795 (PMC6933328; doi:10.1111/jcmm.14795)
Supplement: Supplementary file 1 [file JCMM-24-850-s001.docx]

**Supplemental Table 1. Heart rate**

| **Heart rate (bpm)** | **Sham** | **Sham+DEX** | **IR** | **I/R+IOX2** | **I/R+DEX** | **I/R+DEX+IOX2** | **I/R+DEX+vehicle** | |
| --- | --- | --- | --- | --- | --- | --- | --- | --- |
| Baseline | 375.5±6.7 | 381.6±7.5 | 373.8±5.9 | 378.8±6.0 | 381.4±6.9 | 382.7±6.0 | | 378.4±9.5 |
| Ischemia 15 min | 377.2±7.8 | 371.9±9.2 | 387.3±5.5 | 374.8±4.9 | 372.5±7.5 | 381.5±9.0 | | 373.6±6.1 |
| Ischemia 30 min | 370.3±10.5 | 368.7±8.8 | 381.2±11.1 | 375. 7±5.7 | 370.2±9.2 | 384.3±6.5 | | 379.1±11.0 |
| Reperfusion 15 min | 375.2±6.7 | 366.1±7.6 | 376.2±5.4 | 370.5±5.9 | 371.3±6.3 | 369.3±9.1 | | 368.3±7.9 |
| Reperfusion 30 min | 378.7±8.8 | 369.3±7.8 | 380.0±6.4 | 378.8±4.7 | 369.2±5.5 | 371.3±7.4 | | 372.3±6.4 |
| Reperfusion 60 min | 374.5±10.8 | 370.5±9.3 | 379.8±4.1 | 376.7±6.8 | 364.9±10.2 | 370.3±7.0 | | 366.8±8.9 |
| Area Under Curve | 1876±33.5 | 1832±32.7 | 1902±27.4 | 1869±20.2 | 1843±28.7 | 1887±29.9 | | 1851±27.5 |

n = 6. Data are shown as means ± SEM. DEX, dexmedetomidine; I/R, ischemia/reperfusion.

**Supplemental Table 2. Blood analysis at 6 h of reperfusion**

|  | **Sham** | **Sham+DEX** | **I/R** | **I/R+IOX2** | **I/R+DEX** | **I/R+DEX+IOX2** | **I/R+DEX+vehicle** |
| --- | --- | --- | --- | --- | --- | --- | --- |
| pH | 7.39±0.02 | 7.37±0.02 | 7.38±0.02 | 7.40±0.02 | 7.40±0.02 | 7.41±0.02 | 7.37±0.02 |
| PaO_2_ (mmHg) | 73.34±1.30 | 72.11±1.33 | 72.47±1.29 | 71.80±0.86 | 71.91±0.88 | 70.93±1.00 | 71.77±0.86 |
| PaCO_2_ (mmHg) | 37.37±1.12 | 31.39±1.84 | 30.86±1.53 | 35.07±1.77 | 33.14±1.83 | 34.31±2.15 | 32.89±1.14 |
| SaO_2_ (mmHg) | 94.16±0.64 | 92.46±0.80 | 91.73±0.83 | 91.66±0.96 | 92.29±0.84 | 92.54±1.12 | 92.87±0.81 |
| Hb (g/dL) | 12.86±0.31 | 12.09±0.55 | 12.84±0.37 | 13.06±0.72 | 12.76±0.52 | 13.20±0.42 | 12.11±0.45 |
| Hct (%) | 40.7±0.9 | 37.0±1.6 | 39.4±1.2 | 41.9±1.8 | 37.3±1.6 | 40.9±1.3 | 40.4±0.8 |
| Na^+^ (mmol/L) | 141.6±0.9 | 143.1±1.1 | 143.1±1.9 | 142.3±1.4 | 142.4±1.6 | 143.1±1.0 | 142.6±1.6 |
| K^+^ (mmol/L) | 4.30±0.18 | 4.34±0.26 | 4.74±0.31 | 4.53±0.16 | 4.43±0.09 | 4.48±0.31 | 4.20±0.14 |
| Ca^2+^ (mmol/L) | 1.10±0.03 | 1.07±0.04 | 1.04±0.01 | 1.13±0.04 | 1.10±0.03 | 1.10±0.05 | 1.08±0.04 |
| Cl^-^ (mmol/L) | 102.7±1.3 | 106.3±2.1 | 106.3±2.7 | 105.0±1.7 | 103.1±1.8 | 104.9±1.9 | 104.7±1.6 |
| HCO_3_^-^ (mmol/L) | 25.16±0.34 | 21.87±1.24 | 21.5±0.91 | 22.74±0.97 | 21.61±0.53 | 21.91±0.90 | 21.76±0.73 |
| BE (mmol/L) | 0.73±0.89 | -1.56±1.06 | -1.73±0.97 | -0.70±1.05 | -1.06±1.09 | -2.16±1.42 | -1.47±1.31 |

n = 6. Data are shown as means ± SEM. DEX, dexmedetomidine; I/R, ischemia/reperfusion.
